# Simple Diet the Safest

**Published:** 1891-07

**Authors:** 


### SIMPLE DIET THE SAFEST.

Yes, the simplest is the safest, and the best is the cheapest. The butcher, for example, or the egg merchant, cannot adulterate his wares, but he may have several qualities ; and there is a stage in respect to all animal foods which renders them to a large extent poisonous, and this is as bad, if not worse than adulteration.

We often hear it said that shop eggs, as they are called, are good enough for frying, with bacon or ham. This is a mistake; an egg that has even a suspicion of staleness about it is deleterious to health, not to say dangerous, no matter whether it be fried or boiled. The same may be said of flesh meats of all kinds.

It is a truism that any kind of food or mixed diet which requires the aid of stimulant for their digestion is not salutary. Such a diet is a tax upon the whole system, and causes heat and discomfort, and a feverish state of the blood, which can only end in debility of the nervous system and more or less prostration.

Those who would obtain the greatest amount of health and comfort from the food they eat must be most careful in its selection. Every one ought to know what agrees with him and what does not. Through the summer months the table should be abundantly supplied with fruits and vegetables. Children especially should be fed liberally on fruits, to avoid heat-rashes, rushes of the blood to the head, and digestive troubles. As a rule the healthy stomach will easily absorb any reasonable quantity of fruit, taken at proper seasons. Weak and abused digestive organs will have to be treated more carefully, but will ultimately grow accustomed to a fruit diet and be all the better for it. To do good, fruit must be properly ripe and sound. If eaten green or between meals it will interfere with digestion and cause bowel troubles. Acids prevent calcarous degeneration, keeping the bones elastic, as well as preventing the accumulation of earthy matters. This is because of their solvent power. Fruit is perfect food when fully ripe, and if it were in daily use from youth to age, there would be less gout, gallstones and stone in the bladder.

